# Protocol for making an animal model of “blindsight” in macaque monkeys

**DOI:** 10.1016/j.xpro.2022.101960

**Published:** 2022-12-23

**Authors:** Norihiro Takakuwa, Kaoru Isa, Reona Yamaguchi, Hirotaka Onoe, Jun Takahashi, Masatoshi Yoshida, Tadashi Isa

**Affiliations:** 1 Department of Neuroscience, Graduate School of Medicine, Kyoto University, Kyoto 606-8501, Japan; 2 Max Planck Institute for Brain Research, 60438 Frankfurt am Main, Germany; 3 Institute for the Advanced Study of Human Biology (WPI-ASHBi), Kyoto University, Kyoto 606-8501, Japan; 4 Human Brain Research Center, Graduate School of Medicine, Kyoto University, Kyoto 606-8507, Japan; 5 Department of Clinical Application, Center for iPS Cell Research and Application, Kyoto University, Kyoto 606-8507, Japan; 6 Center for Human Nature, Artificial Intelligence, and Neuroscience (CHAIN), Hokkaido University, Sapporo, Japan

**Keywords:** Microscopy, Model Organisms, Neuroscience, Cognitive Neuroscience, Behavior

## Abstract

Patients with damage to the primary visual cortex (V1) can respond correctly to visual stimuli in their lesion-affected visual field above the chance level, an ability named blindsight. Here, we present a protocol for making an animal model of blindsight in macaque monkeys. We describe the steps to perform pre-lesion training of monkeys on a visual task, followed by lesion surgery, post-lesion training, and evaluation of blindsight. This animal model can be used to investigate the source of visual awareness.

For complete details on the use and execution of this protocol, please refer to Yoshida et al. (2008)^1^ and Takakuwa et al. (2021).^2^

## Highlights

• A protocol for making a "blindsight" model in macaque moneys
• Lesion surgery to remove most parts of V1 by aspiration
• Train monkeys and evaluate task performance to assess "blindsight" ability
• Post-mortem assessment to confirm the extent of V1 lesion and influence in the LGN

## Abstract (editor highlights)

Publisher’s note: Undertaking any experimental protocol requires adherence to local institutional guidelines for laboratory safety and ethics.

## Graphical abstract

**Figure:**
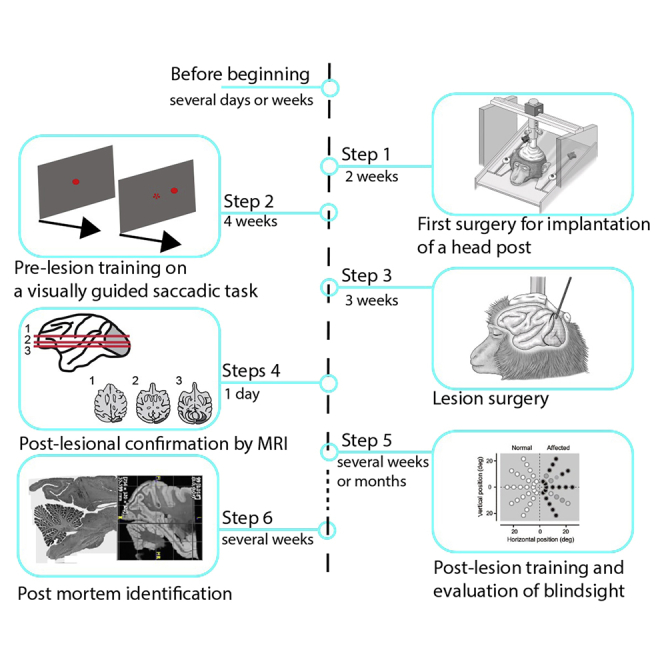

## Before you begin

We use Japanese macaque monkeys (*Macaca fuscata*) as the model animal for blindsight. In order to use macaque monkeys, it is essential to have a housing environment that strictly fulfills the animal welfare criteria described in the laws or guidelines of your country.

The following guidelines will be helpful when you start breeding primates:

 Guide for the Care and Use of Laboratory Animals (https://grants.nih.gov/grants/olaw/guide-for-the-care-and-use-of-laboratory-animals.pdf).

 NC3Rs guidelines: non-human primate accommodation, care, and use (https://nc3rs.org.uk/non-human-primate-accommodation-care-and-use).

 Guidelines for the Care and Use of Nonhuman Primates of the Japan Neuroscience Society (https://www.jnss.org/en/animal_primates).

Your institutional committee for the animal experimentation is responsible for confirming that your research plan is in accordance with the guidelines in your country or region. The institutional permissions are mandatory for starting your experiments.

***Note:*** In our case, all of the experimental procedures were approved by the Committees for Animal Experiments at the Graduate School of Medicine in Kyoto University.

Since macaque monkeys are highly susceptible to tuberculosis, it is desirable for users to take epidemic prevention measures such as confirming that the animals are not affected by tuberculosis with chest X-ray examinations and/or interferon gamma release tests. In addition, it is desirable that the users confirm that the animals have sufficiently high measles antibody levels.

The monkeys have to receive B-virus and simian retrovirus (SRV) antibody inspections. If an animal is kept for longer than 1 year, it is strongly recommended that regular health examinations including tuberculin, dysentery, and salmonella tests are performed.

Newly received animals should be given a period for acclimation after quarantine, before starting the experimental procedures.^3^

We use a monkey chair when conducting behavioral recordings. Habituation to the monkey chair is required. These steps are largely dependent on your monkey’s housing environment. Please confirm the guidelines of your country and choose suitable environments and methods to meet them.

After satisfying all of these conditions, you can start the experimental protocol that is described in this paper.

### Baseline magnetic resonance imaging (MRI)

**Timing: 1 day**

**Figure 1:**
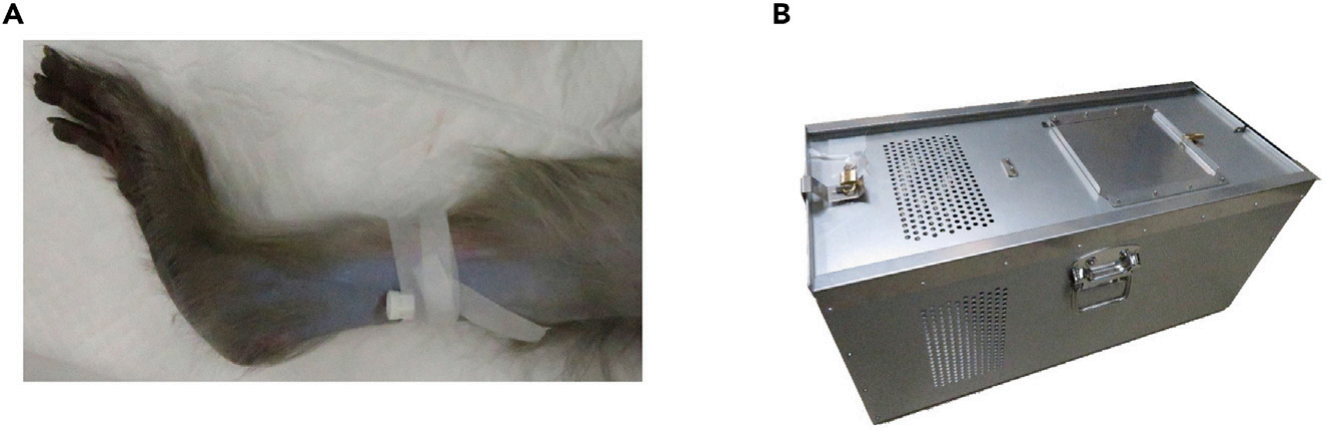
Preparation for surgery (A) The indwelling needle is fixed with surgical tape after being inserted into the vein. (B) A carry box for a monkey should have holes for ventilation and two locks.
  c. Fix the indwelling needle and tube using surgical tape. The surgical tape should cover the connection between the needle and tube.
  d. Intubation of the trachea to secure the airway.
    i. Visualize the trachea using a laryngoscope by moving the monkey’s head dorsally.
    ii. Insert the tracheal tube into the trachea and inflate the cuff of the tube.
      ***Note:*** The size of the tube should be checked carefully in advance considering the body size of the monkey.
  e. Put the monkey in a double-locked carry box and take it to the MRI room (Figure 1B).
3. Take MR and computed tomography (CT) images.
  a. Fix the monkey’s head to MR-compatible stereotaxic apparatus.
  b. Anesthetize the animal with inhalation of isoflurane (1.0%–2.0%).
  c. Monitor SpO_2_, heart rate, and pCO_2_ during imaging.
  d. Warm the animal’s body using a microwavable heating pad to keep body temperature above 36°C.
  e. Obtain high-resolution 3D T1- and T2-weighted whole brain images through a 3D fast spin-echo sequence with two echoes in a 3-Tesla MRI system (Verio; Siemens, Munich, Germany) using a custom-built birdcage radio frequency coil with a 10 cm inner diameter (Takashima Seisakusho Co., Ltd., Tokyo, Japan), and acquire brain CT images with a 320-detector-row CT scanner (Aquilion ONE; Toshiba Medical Systems Corporation, Tochigi, Japan).
  f. Confirm there are no defects in the brain. Align the MR images to the subject’s CT images by applying a linear, rigid coregistration algorithm (PMOD Technologies, Zürich, Switzerland).

***Note:*** The merged images will be used for subsequent surgeries to decide where to aspirate the visual cortex and where to place the recording chamber.

1. Anesthetize the monkey with ketamine, medetomidine, and midazolam in its cage.
  a. Food restriction. The monkey should not eat anything for 24 h before anesthetization.
  b. Restrain the monkey in its cage. In our monkey cage, the backside wall can be pulled to the front to restrain the animal.
  c. Inject ketamine (1 mg/kg), medetomidine (0.04 mg/kg), and midazolam (0.3 mg/kg) into the lateral thigh muscle. If the monkey is not sufficiently restrained, it may extend its hands to us or the injection syringe. Be careful of its hands.
  d. After the injections, push the cage wall back to create a small space. If the space is too large, the monkey can fall down and be injured. It is preferable that the monkey can lean against the walls of its cage while it is anesthetized.
  e. Put the monkey into a carry box and take it to the preparatory room. The carry box should have air holes. Double-lock the lid when carrying the monkey.
2. Secure the venous line and airway.
  a. Clean the skin of the lateral regio suralis with 70% ethanol after removing the hair with a shaver.
  b. Insert an indwelling needle into the vein in the lateral regio suralis.
    ***Note:*** Push the proximal part of the injection site below the knee to better visualize the vein. Attach an injection port cap (Figure 1A). This venous line is used in emergency situations, e.g., heart rate drop.

### Behavioral testing apparatus

**Timing: several days or weeks**

**Figure 2:**
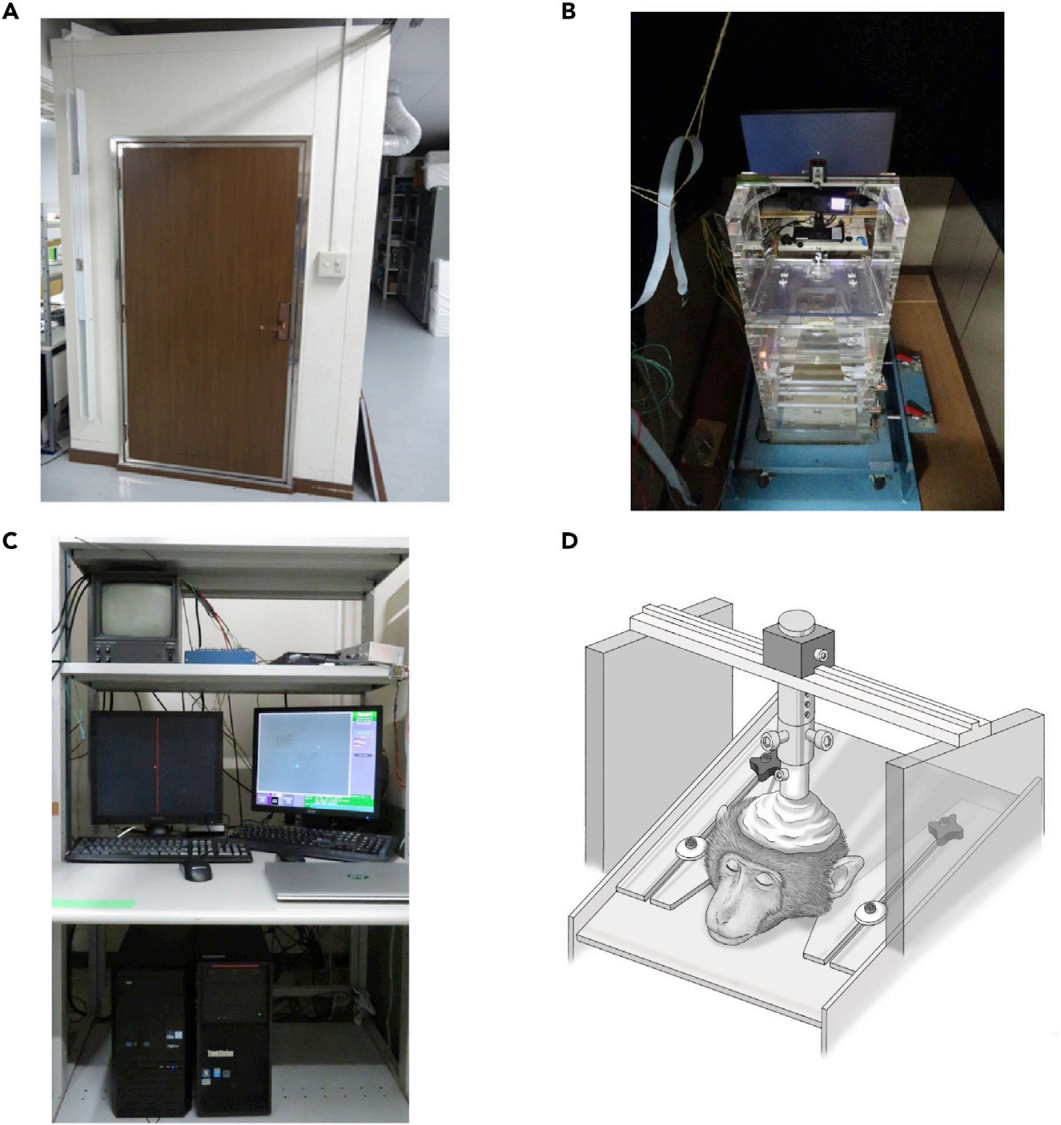
The monkey chair and recording environment (A) All experiments are conducted in a soundproof booth. (B) The chair is fixed by a fixing plate on the floor, and a display for plotting tasks is placed at the back of the booth. (C) Recording systems located outside the soundproof booth. Researchers always monitor eye movements by an eye tracker, record eye position and correct windows, a video of the monkey in the soundproof booth, and plots of the task. (D) The head is fixed to the monkey chair when the monkey performs an eye movement task.

***Note:*** Monkeys are very sensitive to sounds if they are not trained to ignore them. A soundproof booth can help the monkeys maintain steady task performance.

4. Use a soundproof booth for behavioral tests on the monkeys before and after lesioning of the primary visual cortex (V1) (Figure 2A).

5. Task control system.
  a. Use Psychophysics Toolbox version 3 (PTB-3; http://psychtoolbox.org/) on MATLAB (MathWorks) for stimulus plotting and data recording. Control the parameters of the visual stimulus as described below. For example, present a red dot with a radius of 1.3° and luminance contrast of 0.9. The locations of the visual stimulus are determined based on the distance between the monitor screen (Diamondcrysta WIDE RDT272WX [BK]; Mitsubishi) and the monkey’s eyes.
    ***Note:*** In our case, the distance is 60 cm, with which 10° in the view corresponds to 340 pixels on the screen. In addition, it is necessary to decide the light level in the soundproof booth, because it also affects the luminance contrast of the visual stimulus (Figure 2B).
  b. Confirm the time delay between command and visual stimulus presentation using a photoLED.
    i. Attach the photoLED at the location where the visual stimulus will be presented on the screen, and record the signal of the photoLED with the time of the command in MATLAB to plot the visual stimulus.
    ii. The difference of time between the command and photoLED signal indicates the delay, which must be consistent, and has to be taken into account during data analysis.
  c. Prepare the eye tracking system. Record eye movements using an eye tracker (EyeLink 1000 PLUS; SR Research, Ottawa, Canada) at 1,000 Hz. The gaze data are also used for task control in Psychophysics Toolbox version 3 (Figure 2C).
  d. Prepare the reward system. In our case, water is used as a reward. The amount is controlled by the opening and closing of a valve. Approximately 0.2 mL reward is delivered following one correct trial, and the monkey receives 100–300 mL in a daily experiment.

## Key resources table

**Table:** 

REAGENT or RESOURCE	SOURCE	IDENTIFIER
**Antibodies**		

Mouse monoclonal anti-NeuN antibody (1:200)	Millipore	#MAB377
Biotin goat anti-mouse IgG (1:200–500)	Vector Laboratories	#BA9200
mouse monoclonal anti-calmodulin-dependent protein kinase II alpha antibody (1:100–1000)	Invitrogen (ThermoFisher)	#MA-1-048

**Chemicals, peptides, and recombinant proteins**		

Ketamine	Daiichi Sankyo Propharma Co., Ltd.	D07711
Medetomidine	Nippon Zenyaku Kogyo Co., Ltd.	D04883
Midazolam	Maruishi Pharmaceutical Co., Ltd.	D05500
Isoflurane	Mylan Seiyaku Co., Ltd.	D00545
Soldem 3A infusion	Terumo Corporation	YJ code: 3319510A5098
Ketoprofen	Nissin Pharmaceutical Co. Ltd.	D00132
Cefazolin sodium	Nichi-Iko Pharmaceutical Co., Ltd.	D00905
Mannitol	Yoshindo, Inc.	D00062
Xylen	Nacalai tesque, Inc.	36612-35
Cresyl violet solution	WALDECK	1A-400
Mounting medium	Sigma	06622-100ML
Triton X-100	Sigma	T8787-100ML
Normal goat serum	Vector Laboratories	#S-1000
ABC-Elite solution	Vector Laboratories	#PK-6100
Ammonium nickel (Ⅱ) sulfate hexahydrate	Fujifilm Wako Purechemical Co.	146-01012
Acetic acid	Fujifilm Wako Purechemical Co.	012-00245
Sodium acetate trihydrate	Fujifilm Wako Purechemical Co.	198-01055
Sodium chloride	Nacalai tesque Co.	31319-45
Di-sodium hydrogenphosphate	Nacalai tesque Co.	31723-35
Sodium dihydrogenphosphate dihydrate	Fujifilm Wako Purechemical Co.	192-02815
Tris base	Sigma	T6791-500G
Tris hydrochloride	Sigma	T5941-1KG
Hydrogen peroxide	Nacalai tesque Co.	20779-65
Carrageenan lambda	Sigma	22049-6G-F
Fish gelatin	Sigma-Aldrich	G7765
Sodium azide	Fujifilm Wako Purechemical Co.	195-11092
DAB tablet	Fujifilm Wako Purechemical Co.	040-27001
Nickel(II) chloride hexahydrate	Sigma-Aldrich	227676-500G

**Experimental models: Organisms/strains**		

Japanese macaque (*Macaca fuscata*)∗	National Bioresource Project, MEXT, Japan	N/A

**Software and algorithms**		

MATLAB	MathWorks	Licensed
Psychophysics Toolbox version3	https://github.com/Psychtoolbox-3/Psychtoolbox-3/tree/3.0.18.12	http://psychtoolbox.org/
A linear, grid coregistration algorithm	PMOD Technologies	https://www.pmod.com/web/
Imaging software	PMOD Technologies	https://www.pmod.com/web/
Fiji	(Schindelin et al.)^4^	https://github.com/fiji/fij

**Other**		

tracheal tube	Standard endotracheal tube, with cuff, Murphy type	224AABZX00164000
Indwelling needle	Terumo co., Ltd	SR-OT2419C
Surgical tape	Nichiban Co., Ltd	12-9, 4987167004132
laryngoscope	Muromachi Kikai Co. Ltd.	MK-FOHA06, MK-STMA22
biomonitor	Nihon Kohden Co.	BMS-3592
perforated drape	Japan Medical Produucts Co., Ltd.	RH-22C60-PP
Micro grinder	Minitor Co., Ltd.	Minimo One C2012
Dental resin	GC Corporation	UnifastII A3
Reinforced plastic screws	RENY Cross Hole (+) Knurled Knob Screws	CSPPNR-RENY-M3-4
Titanium screw 2.7 mm × 6 mm	DePuy Synthes J&J	402-006
Manipulator	Narishige	SM-15
Dental drill	Minitor Co., Ltd	Minimo Ver.2
Bipolar coagulator	KIRIKAN Ltd.	ADC140
Aspiration pump	Shin-Ei Industries, Inc.	Minic W-II
Frazier (suction catheter)	Japan FRIGZ Medico	φIGZ-5.0 × 230 mm Z186-X158
Absorbable hemostatic gelatin sponge	LTL Pharma	Spongel
Isoflurane anesthetic vaporizer	KimuraMed	KIV-3
7-0 polypropylene suturing thread	Akiyama Medical MFG.co., Ltd.	EN007
Artificial dura mater	TamaBio Co.	Durabeam
Peristaltic pump	Cole-Parmer Instrument Co.	Model 07554-80
EyeLink 1000 PLUS	SR Research	https://www.sr-research.com/hardware/
Monitor screen	Mitsubishi	Diamondcrysta WIDE RDT272WX[BK]
Valve (solenoid valve)	CKD corporation	USB3-6-2-DC12V
3-Tesla MRI system	Siemens	Verio
Birdcage radio frequency coil	Takashima Seisakusho Co.	Special order
CT scanner	Toshiba Medical System Co.	Aquilion ONE
Proton-free magnetic susceptibility matching fluid	3M Co.	Fluorinert FC-43
Investigational whole-body human 7T scanner	Siemens Healthineers	MAGBETOM 7T
Light microscope	Keyence	BZ-X710
Light microscope	Keyence	BZ-X810
Carry box	Gifu Plastic Industry Co., Ltd.	RVBox, 702 × 370 × 390 mm
Soundproof booth	Kawai	(Special Order)
Monkey cage	Tokiwa Kagaku Kikai Ltd.	(Special Order)
Monkey chair	O’Hara & Co., Ltd.	(Special order)
Microwavable heating pad	Hakugen Earth Co., Ltd	# 330468
Head post	Unique Medical Co	OM218-006
Autoclave	Canon Lifecare Solutions Co.	MAC-580
Gas sterlization	Canon Lifecare Solutions Co.	SAH-160

∗; Wild type Japanese macaques are used in this protocol. Sex and age do not matter, but young monkeys tend to recover faster after the V1 lesion surgery than old ones.

## Materials and equipment

Here, we show solution recipes that are used at steps 44–46 in “post-lesion training and evaluation of blindsight” section.

**Table:** 0.02 M Acetate buffer pH3.8 (Histology 1)

Reagent	Final concentration	Amount
Acetic acid	0.02N	0.23 mL
Sodium acetate trihydrate	0.02N	8.16 g
Water	N/A	Up to 500 mL
**Total**	**N/A**	**500 mL**

[Note on storage conditions] The pH of the solution is adjusted with 1N HCL and can be stably stored at room temperature (15°C–25°C) for 1 week.

**Table:** Cresyl violet solution (Histology 1)

Reagent	Final concentration	Amount
0.5% Cresyl Violet	0.1%	100 mL
0.02 M Acetate buffer pH3.8	16 mM	400 mL
**Total**	**N/A**	**500 mL**

[Note on storage conditions] Solution should be prepared at least 1 week before usage.

And they can be stored at room temperature (15°C–25°C) for 3 months. The pH of the solution is important.

**Table:** 0.05 M PBS (Histology 2,3)

Reagent	Final concentration	Amount
Sodium Chloride	0.9%	8.99 g
Di-sodium hydrogenphosphate	0.05 M	14.326 g
Sodium dihydrogenphosphate dihydrate	0.05 M	1.560 g
Water	N/A	Up to 1,000 mL
**Total**	**N/A**	**1,000 mL**

[Note on storage conditions] Solution can be stably stored at room temperature (15°C–25°C) for 1 week.

**Table:** Blocking solution (Histology 2)

Reagent	Final concentration	Amount
0.05 M PBS	0.05 M	89.5 mL
20% Triton X-100	0.1%	0.5 mL
normal goat serum	10%	10 mL
**Total**	**N/A**	**100 mL**

[Note on storage conditions] The blocking solution should be fleshly prepared or should be frozen at -20 degree and stored for 2 month. The usage of Triton X100 is important.

**Table:** 0.05 M TBS (Histology 2,3)

Reagent	Final concentration	Amount
Sodium Chloride	0.9%	8.77 g
Trizma-base	0.05 M	1.39 g
Tris hydrochloride	0.05 M	6.06 g
Water	N/A	Up to 1,000 mL
**Total**	**N/A**	**1,000 mL**

[Note on storage conditions] Solution can be stably stored at room temperature (15°C–25°C) for 1 week.

**Table:** DAB staining solution (Histology 2)

Reagent	Final concentration	Amount
DAB tablet (5 mg)	0.01%	2 pieces
ammonium nickel (II) sulfate hexahydrate	1.0%	1.0g
0.05 M TBS	0.05 M	99 mL
0.1% Hydrogen peroxide	0.0003%	0.3 mL
**Total**	**N/A**	**100 mL**

[Note on storage conditions] The DAB tablet should be newly dissolved and the solution should be filtrated.

**Table:** Blocking solution (Histology 3)

Reagent	Final concentration	Amount
0.05 M PBS	0.05 M	97 mL
20% Triton X-100	0.1%	0.5 mL
normal goat serum	2%	2 mL
Fish gelatin	0.5%	0.5 mL
Carrageenan lambda	0.5%	0.5 g
Sodium azide	0.02%	0.02 g
**Total**	**N/A**	**100 mL**

[Note on storage conditions] The blocking solution should be fleshly prepared or should be frozen at -20 degree and stored for 1 month.

**Table:** DAB staining solution (Histology 3)

Reagent	Final concentration	Amount
DAB tablet (5 mg)	0.04%	8 pieces
8% Nickel(II) chloride	0.04%	0.5 mL
0.1 M Tris buffered saline	0.1 M	99 mL
0.1% Hydrogen peroxide	< 0.003%	< 3 mL
**Total**	**N/A**	**100 mL**

[Note on storage conditions] The DAB tablet should be fleshly dissolved and filtration is needed before usage. Addition of the hydrogen peroxide after pre-incubation should be carefully controlled under the microscope.

## Step-by-step method details

### First surgery for implantation of a head post

**Timing: 2 weeks to complete all surgical procedures**

A head post is attached to the center of the monkey’s skull to fixate its head to the monkey chair during behavioral recording (Figure 2D). Here, we describe how the head post is implanted on the monkey’s head.

1. Decide the location of the head post before surgery.

***Note:*** The head post should be located at a position that does not interfere with future surgeries (i.e., V1 lesioning and recording chamber placement). In our case, the monkey’s head is fixed with a single pole (Figure 2D), so the head post should be implanted at approximately the center of the skull (AP 6–10 mm in the stereotaxic coordinates of Paxinos et al. (2009)).^5^

2. In advance of the day of surgery.
  a. Food restriction. The monkey should not eat anything for 24 h before surgery.
  b. Clean the surgery room. Equipment in the surgery room should be cleaned by wiping with ethanol or hypochlorous acid.
  c. Sterilize the tools for surgery. All surgery tools should be cleaned and sterilized by either autoclave (MAC-580; Canon Lifecare Solutions Co., Ltd., Kanagawa, Japan) or gas sterilization (SAH-160; Canon Lifecare Solutions Co., Ltd., Kanagawa, Japan).
3. Introduce anesthesia. This step is the same as step 1 in the section “baseline magnetic resonance imaging (MRI).”
4. Preparation in the surgery room.
  a. Remove the monkey’s hair around the head with depilatory cream and the lateral regio suralis with a shaver.
  b. Insert an indwelling needle into the vein in the lateral regio suralis after sterilizing the area with 70% alcohol.
  c. Connect the indwelling needle to a tube that is connected to a syringe filled with extracellular fluid replacement solution (Soldem 3A).
  d. Inject ketoprofen (2.0 mg/kg, also after surgery and the next morning, if needed) and cefazolin sodium (30 mg/kg) intramuscularly (mainly to the hamstring muscles).
5. Tracheal intubation. See 2-d in the “baseline magnetic resonance imaging (MRI)” section.
6. Connect the biomonitor (BSM-3592; Nihon Kohden Co., Ltd., Tokyo, Japan). Connect the tracheal tube to Isoflurane anesthetic vaporizer (KIV-3, KimuraMed, Tokyo, Japan). Administer isoflurane at 1.0%–2.0%. Monitor rectal temperature, electrocardiogram, respiration, SpO_2_, isoflurane concentration during inhalation and exhalation, and pulse rate during surgery.
7. Fix the head to the head stage.
8. Clean the monkey’s head first with povidone iodine and then with 70% ethanol.
9. Wash the operator’s hands using chlorhexidine gluconate solution and povidone iodine. The operator should wear a sterilized gown, gloves, mask, and cap.
10. Cover the monkey’s body with a perforated drape, except for the head.
11. Open the skin of the head in the center along the rostral-caudal axis.

***Note:*** If you cannot secure a sufficient surgical field, a part of the skin should be removed. Remove all tissue on the skull to secure the surgical field.

12. Attach the screws to the skull. To stabilize the dental resin that covers the surgical field to fix the head post, we implant 8–10 reinforced plastic screws and titanium screws as anchors.

***Note:*** Make holes for individual screws by using a dental drill, make threaded screw holes with a tap (diameter: 2.2 mm), and then fix the screws with a screwdriver.

***Note:*** After implantation, check the stability of the screws.

13. Fix a head post. Move the head post to the location you decided using a manipulator. Fix the head post with dental resin. After confirming that the resin has hardened, take off the manipulator. Suture the skin to avoid exposing the inner surface of the skin.
14. Stop isoflurane administration. Remove the indwelling needle and tracheal tube, and prepare to take the monkey back to its cage. It is recommended that the biomonitor should be observed including SpO_2_ until the monkey fully awakes.
15. After surgery, allow at least 1 week for the monkey to recover.

### Pre-lesion training on a visually guided saccadic task

**Timing: 4 weeks**

**Figure 3:**
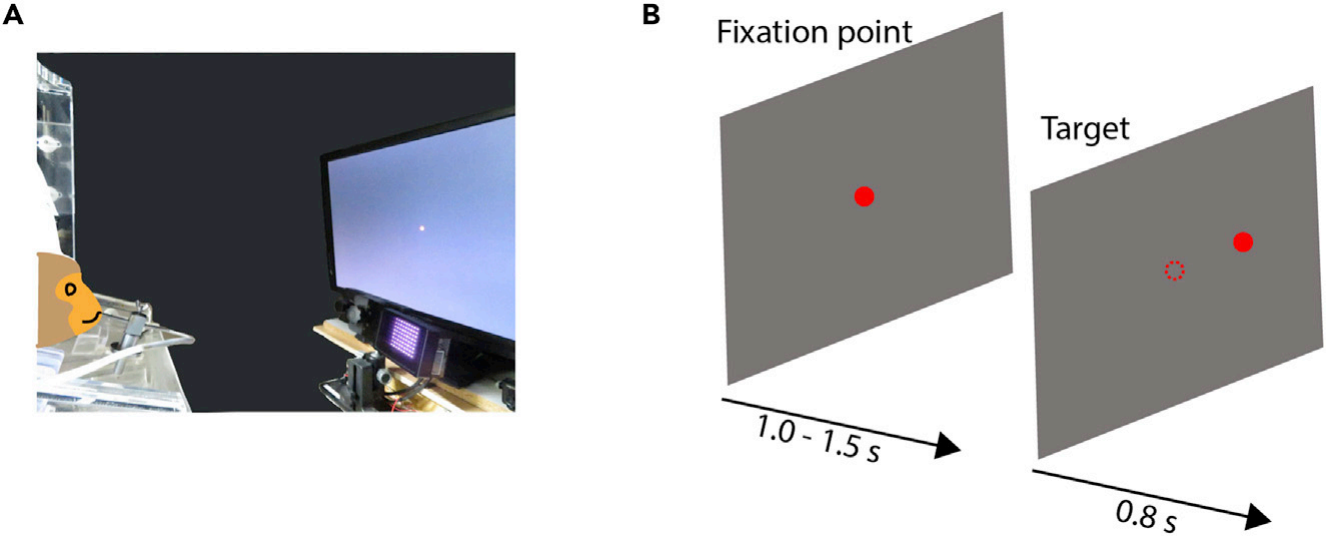
Visually guided saccadic task (A) The monkey is placed in front of a monitor and performs the visually guided saccadic task. A tip of a reward tube is put inside the monkey’s mouth. If the monkey performs eye movements correctly, a water reward is delivered. (B) A scheme of the visually guided saccadic eye movement task. At first, the monkey is required to fixate its gaze on the center red dot. Second, the monkey has to move its gaze to the peripheral dot. A dashed circle on the right panel indicates the location of the fixation point. The central fixation point is completely eliminated when the peripheral dot is appeared.

Here, the monkey learns a visually guided saccadic task (Figure 3) in which they have to move their gaze toward a visual target on the screen from a central fixation point. The performance of visually guided saccadic eye movements allows us to confirm whether the V1-lesioning surgery is conducted properly.

16. Start water restriction.
  a. Restrict water intake from 1 day before training.

***Note:*** Determine the total amount of daily water supply for each monkey according to the guidelines and if the water delivered during the experiment did not reach this amount, provide the remaining volume of water after the experiment.

***Note:*** During water restriction, check the body weight of the monkey every day. In case of a decrease of body weight of more than 20% from the beginning of water restriction, it should be stopped immediately.

17. Familiarization with the head-fixed condition.
  a. Fix the head position by using the head post (Figure 2D).
    ***Note:*** At the beginning, the monkey might dislike its head being fixed. Give food or water to the monkey as a reward immediately after its head is fixed. After giving all the prepared food or water, release the head quickly.
  b. Gradually extend the amount of time the head is fixed for.
    If the monkey does not hesitate to have its head fixed, give food or water at a slower pace. Head fixation for more than 30 min is necessary for the following experiments.
    ***Note:*** Eventually, the monkey has to stay in the soundproof experimental booth in the head-fixed condition during the whole task period, that is, for 2–3 h/day.

18. Training on the visually guided saccadic task.
  a. If water restriction was stopped after the familiarization, start water restriction again on the day before the experiment.
  b. Put the monkey in the experimental booth in the head-fixed condition.
    i. Reduce the light intensity of the room to the level that you decided in advance (see the “behavioral testing apparatus” section).
    ii. Put a reward tube inside the monkey’s mouth. To prevent the liquid in the tube from being emptied by sucking out, make a small vertical cut at the tip of the tube to make air passage.
  c. Calibration of gaze position on the monitor screen. Before the daily experiments, calibration of gaze position is necessary.
    ***Note:*** In our case, the EyeLink 1000 Plus calibration system is used. For calibration, visual stimuli are presented sequentially at different positions on the screen. At each target location and gaze position, the researcher should manually adjust the baseline and gain of the calibrator.
  d. Start with the fixation task.
    i. In this task, present a visual stimulus at the center of the monitor. The size of the stimulus should be large enough for the monkey to respond to it (e.g., 3°) in the first session. If the monkey looks at the visual stimulus, deliver a water reward immediately.
    ii. After the monkey learns the relationship between fixation and reward delivery (the monkey usually learns this association quickly), make the visual stimulus smaller and increase the stay time at the fixation point gradually.
  e. Present a saccadic target in the periphery simultaneously as the fixation point disappears.
    i. The monkey is required to move its gaze toward the saccadic target in less than 700 ms.
    ii. At the beginning, present the target at a single location in the periphery and deliver the reward just when its gaze enters “the target window” (vary target size among the target’s eccentricity, e.g., eccentricity 10°, radius 2.6°).
    iii. Extend the time the monkey has to maintain its gaze in the target window, by which the trial is regarded as correct, as the monkey learns the rules of the task, up to 800 ms.
  f. Increase the number of possible locations at which the saccadic target appears with different directions and different eccentricities relative to the fixation point.
    ***Note:*** In our recent paper,^3^ visual targets were plotted at three eccentricities (5°, 10°, and 15°) × 10 directions (0°, 30°, 60°, 120°, 150°, 180°, 210°, 240°, 300°, and 330°). In one block, five locations were used as possible target locations (single eccentricity and five directions [left or right side]).
    ***Note:*** The correct rate of saccades toward the visual target that is presented at each random point from five possible locations should be higher than 80%. After lesioning V1, the performance level is used as a criterion for the assessment of blindsight.
    **CRITICAL:** If the animal perform worse relative to one day ago, it is possible that the animals confuse what is required. Do not continue the task with the same condition, otherwise the animal starts to hesitate the training (see problem 1).
    ***Note:*** You can start only one new task in each training day. If many new tasks are started together on the same day, it will confuse the monkey and disturb its behavioral learning.

### Lesion surgery

**Timing: 3 weeks to complete all surgical procedures**

Here, we describe how to lesion V1. The majority of V1 is removed by aspiration, except for its rostro-lateral part, which represents the parafoveal region of the retina (0°–2° in eccentricity). After aspiration of V1, a chamber is placed on the skull above the lesioned area for the experimenter to clean the tissue to prevent infection. Steps 20–28 are almost the same as steps 2–10 in “first surgery for implantation of a head post.”

19. Preparation for surgery.

**Figure 4:**
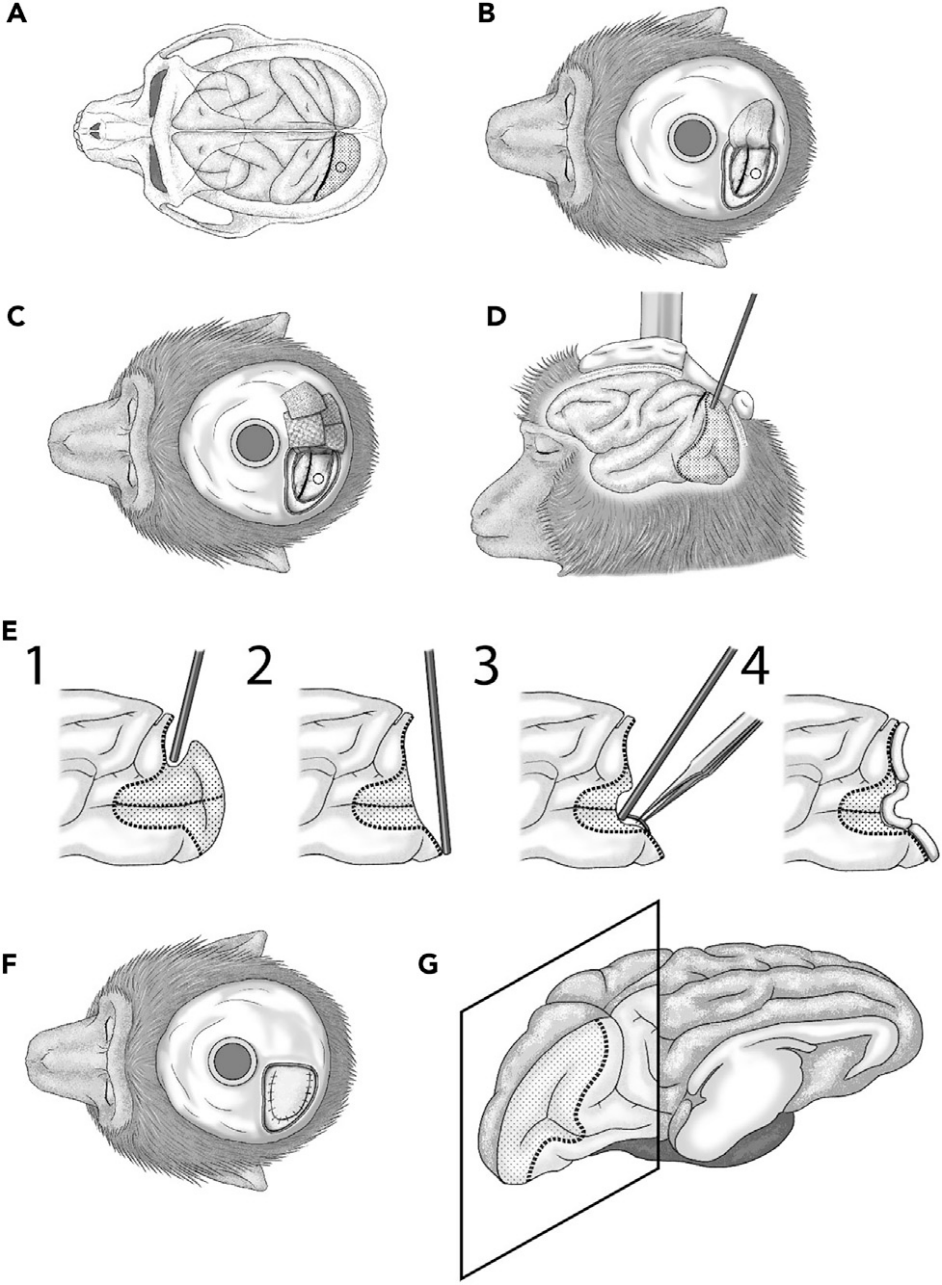
Surgical processes for V1 lesioning (A) V1 is located posterior to the lunate sulcus. Before surgery, the location of the sulcus is determined from MR images. (B) A craniotomy is performed and the dura is cut. The small circle indicates the position of V1. (C) The dura is kept wet with saline-moistened gauze. (D) V1 is removed by aspiration. (E) Specific procedure for V1 aspiration. At first, aspiration is performed from the upper side to the lower side. Second, suction is advanced along the calcarine sulcus. There is a blood vessel in the calcarine sulcus, damage to which is avoided by holding this vessel with forceps. Bleeding can be stopped using absorbable hemostatic gelatin sponges. (F) The dura is then sutured. (G) Left striate cortex illustrated from the medial side. V1 is shown as an area surrounded by a dotted line. The part surrounded by the square shows the coronal section.

Estimate the location of the lunate sulcus as a landmark to judge the rostral border of V1 using the MR images (Figure 4A).

20. Start food restriction, sterilize the surgical tools and clean the surgery room in advance of the day of surgery.
21. Introduce anesthesia and other premedication, and take the monkey to the surgery room. Here, Mannitol (Total 30 mL, 0.2 mL/min) is given through the tube that connects to the indwelling needle.
22. Preparation of the monkey for surgery.
23. Tracheal intubation.
24. Connect the biomonitor to the monkey.
25. Fix the head to the head stage.
26. Clean its head using povidone iodine and ethanol.
27. Wash operator’s hands.
28. Put a perforated drape on the monkey and cover the body, except for its head.
29. Preparation of the aspiration.
  a. Confirm the position of craniotomy (Figure 4A). Use a dental drill to remove the dental resin covering the intended location of the skull opening.
  b. Perform craniotomy. Before craniotomy, exchange the drape and clean the monkey’s head again to remove the powder of the dental resin. The craniotomy should be large enough so the lunate sulcus can be visualized and the suction tube can be inserted.
  c. Make an incision on the dura mater (Figure 4B). Cut the dura in a [U] shape by using spring scissors. Cover the cut end of the dura with saline-soaked gauze to prevent it from drying (Figure 4C).
30. Remove V1 using an aspiration pump with a φ 5.0 mm frazier (Figure 4D).
  a. Before aspiration, coagulate the blood vessels on the brain surface with a bipolar coagulator.
  b. Start aspiration from the top part of the brain to the bottom Take extreme care to prevent bleeding using an absorbable hemostatic gelatin sponge (spongel; LTL Pharma) (Figures 4E1 and 4E2).
31. After that, advance the suction tube along the calcarine fissure to damage the area of V1 representing the peripheral visual field.

***Note:*** There is a blood vessel running along the calcarine fissure (Figure 4E3). To avoid bleeding, hold the vessel with forceps during aspiration.

32. Place an absorbable hemostatic gelatin sponge soaked in Ringer’s solution on the surface of the brain tissue after aspiration to stop bleeding (Figure 4E4). After aspiration, carefully treat the brain tissue with warm Ringer’s solution.

***Note:*** This process is important to prevent infection. Furthermore, carefully observe the remaining brain tissue to confirm whether bleeding is completely stopped.

33. Suture the dura mater (Figure 4F). As the dura is very fragile, be careful not to pull too strongly. We use a 7-0 polypropylene suturing thread. Try to minimize the gap between the cut-end of the dura mater after suturing.
34. After suturing the dura mater, apply artificial dura mater (Durabeam; TamaBio Co., Ltd., Tokyo, Japan) as the surgical closure procedure to prevent postoperative cerebrospinal fluid leak. Attach a chamber to the skull over the sutured dura using dental resin. After surgery, it is necessary to provide care and clean the surface of the lesion area.
35. Stop isoflurane administration.
  a. Remove the indwelling needle, tracheal tube, and prepare to transfer the monkey back to its cage.
  b. Inject ketoprofen (2.0 mg/kg, after surgery and the next morning, if needed), dexamethasone (0.25 mg/kg, for 3 days), and cefazolin sodium (30 mg/kg).

***Note:*** It is recommended that the biomonitor should be observed including SpO_2_ until the monkey fully wakes up.

36. After surgery, allow the monkey to recover for at least 1 week.
37. Keep on taking care of the wounds of the V1-lesioned monkey after surgery. Keep the monkey clean and wash inside the chamber with Ringer’s solution at least once a week.

***Note:*** The general condition of monkeys that experience V1 lesioning is fine and they can move around their cage in the next morning after surgery without apparent paresis or pain. Close monitoring of their behavior should indicate that they have clear visual neglect on the contralesional visual field, where static objects such as food pellets are ignored.

**CRITICAL:** After the lesion, infection sometime occurs. If the chamber fills with cloudy liquid, it is likely infected. In that case, more attention should be paid to physical condition management of monkeys (see problem 2).

### MRI

**Timing: 1 day**

**Figure 5:**
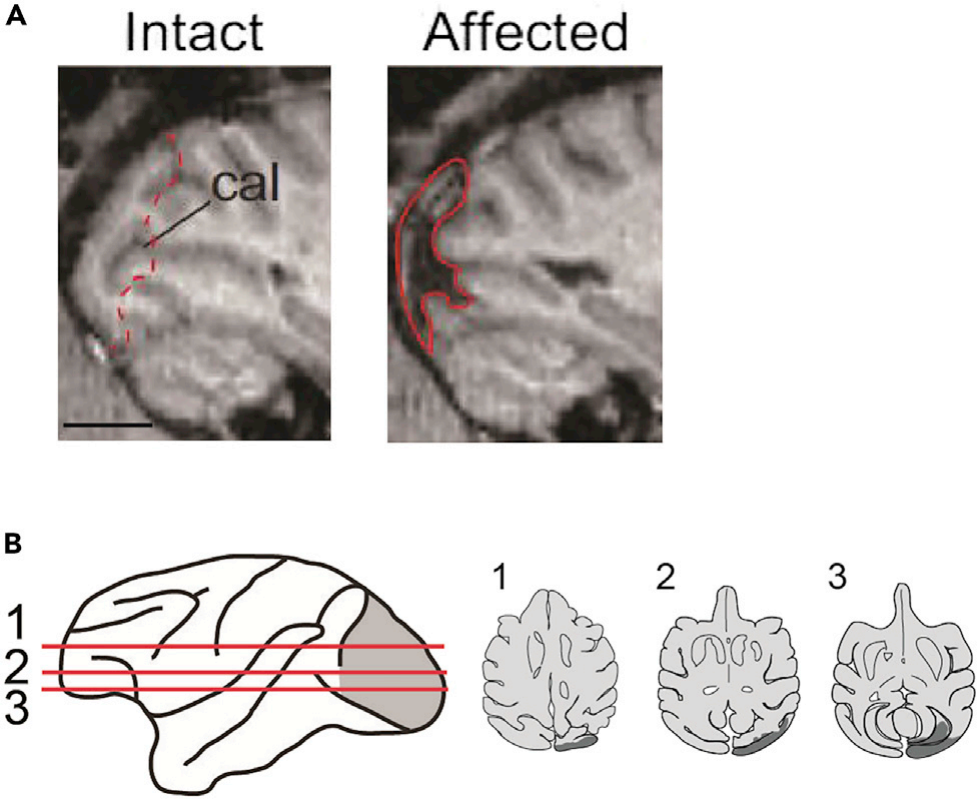
Assessment of the lesion based on MR images (A) The lesioned area is indicated in red on sagittal sections of MR images. Dashed line shows the estimated lesioned area plotted on images of the intact side. The calcarine sulcus (cal) is indicated in the panels of the intact side. Scale bars; A, 10 mm. Modified from Takakuwa et al.^2^ (B) Left: lesioned area (depicted in gray) on a whole brain image. Red lines (1–3) indicate the dorso-ventral levels of horizontal slices shown on the right. Right: lesioned area (depicted in gray) is overlaid in black on axial slices traced from MR images. Modified from Takakuwa et al.^6^

Confirm the lesioned area by taking MR images (Figure 5). The protocols in this section are the same as in the “baseline magnetic resonance imaging (MRI)” section in the “before you begin”.

### Post-lesion training and evaluation of blindsight

**Timing: several weeks or months**

At first, test whether the ability to perform visually guided saccadic eye movements is impaired by lesioning. If impairment is confirmed, start post-lesion training for recovery. Through the training, the monkeys became able to perform the visually guided saccades with more than 80% correct rate toward the high luminance visual targets (> 0.95 Michelson contrast) in the lesion-affected visual field. If the success rate of saccades in different directions with maximum target luminance is saturated during post-lesion training, which usually takes 1–2 months after lesioning,^4^ we start the evaluation of blindsight by plotting the “deficit map” of the monkey. In the deficit map, the sensitivity to luminance contrast is measured systematically for each target location as the detection threshold of the saccadic targets. This deficit map clearly delineates the extent of impairment and helps us understand the lesion-unaffected areas if there is some region of V1 that is spared from damage.

38. Test the ability of saccadic eye movements toward targets presented in the lesion-affected visual field.
  a. Let the monkey perform a visually guided saccadic task that the monkey learned in the pre-lesion training.
    i. First, start with saccadic targets in the intact visual field (ipsilateral to the lesion).
    ii. Eventually, to compare the ability of saccades, present saccadic targets randomly in one of four possible locations. Two are located at the upper and lower positions of the intact visual field, and the others are located at the upper and lower locations of the lesion-affected visual field.

**Figure 6:**
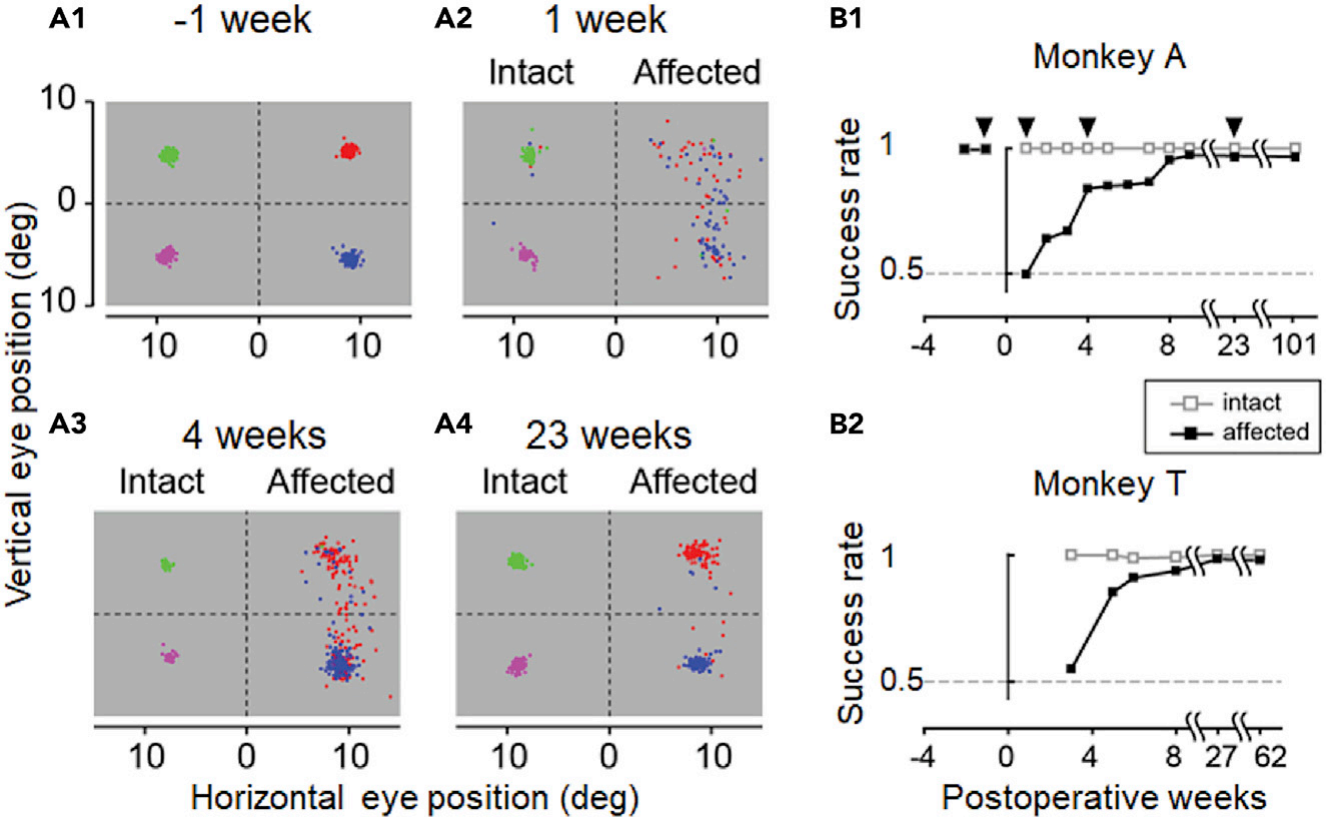
Assessment of the lesion based on behavior (A) End points of saccadic eye movements are plotted at 1 week before (-1) and 1, 4, and 23 weeks after lesioning. After surgery, the monkey loses the ability to make correct visually guided saccadic eye movements toward the lesion-affected visual field. The performance of saccades recovers with approximately 2 months of training. (B) Recovery curves after lesioning in the intact and lesion-affected sides. Modified from Isa and Yoshida.^7^

***Note:*** If the lesion is made as intended, the monkey cannot perform saccades toward the lesion-affected visual field immediately after surgery (Figure 6).

**CRITICAL:** If the monkey can still perform visually guided saccadic eye movements as before lesioning, it is likely that the lesion surgery was unsuccessful. In this case, do not proceed to the next step (see problem 3).

**Figure 7:**
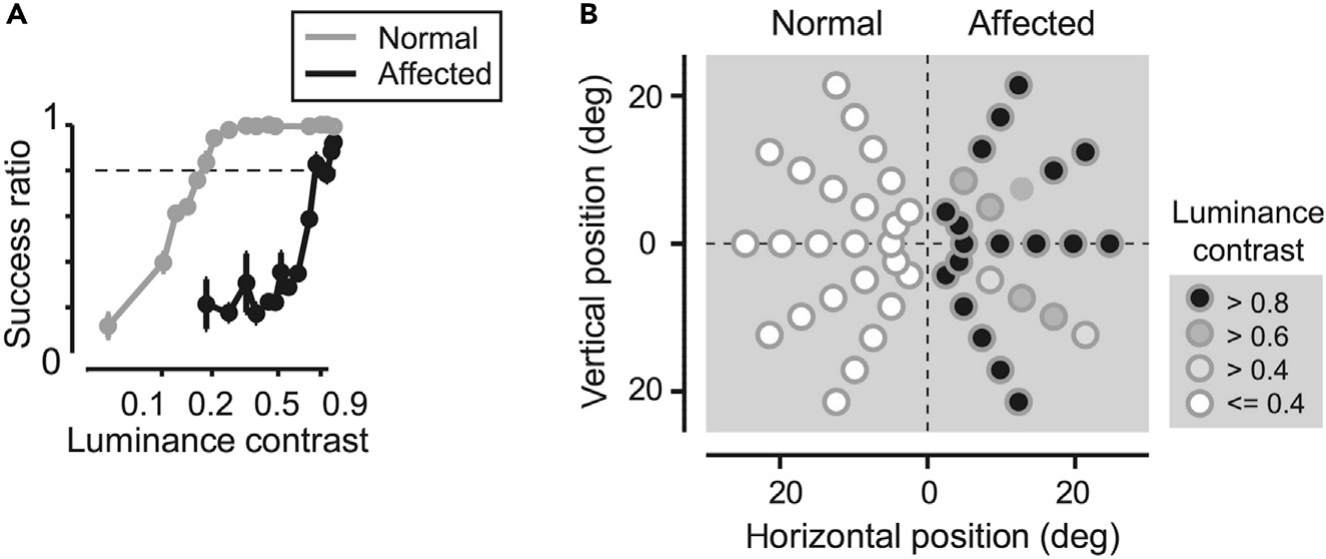
Deficit maps based on sensitivity to luminance contrast (A) Psychometric functions of sensitivity to luminance contrast. The success ratio is plotted against luminance contrast. Gray line, targets in the normal hemifield; black line, targets in the lesion-affected hemifield. Dotted line indicates a success ratio of 0.79, which corresponds to d' = 2 in the five-alternative forced choice method in signal detection theory, used for defining the threshold for luminance contrast. Both plots are fitted with logistic functions with statistical significance (*p* < 0.01). (B) The threshold for luminance contrast is displayed as grayscale for each target position. Modified from Yoshida et al.^1^

39. Training on visually guided saccadic eye movements for recovery.
  a. Train the monkey on a visually guided saccadic task in which a visual target is presented at a single target location in the lesion-affected visual field.
    ***Note:*** During the initial stage of training, it is recommended to start with a large target (∼5° diameter) with high luminance (∼0.95 Michelson contrast) and a longer time window for reaction time (0.5–1.0 s) than in the test.
  b. Increase the number of possible locations that the saccadic target is presented.
  c. Finally, a saccadic target is presented at one of the five possible locations.
  d. To evaluate blindsight, have the monkey perform saccadic eye movements toward targets spanning its entire visual field. For example, three eccentricities (5°, 10°, and 15°) × 10 directions (0°, 30°, 60°, 120°, 150°, 180°, 210°, 240°, 300°, and 330°). In each session, five possible locations are chosen, for example, three eccentricities (10°) × five directions in either the intact or affected hemifield (0°, 30°, 60°, 300°, and 330°).
    ***Note:*** Here confirm that the monkey can perform the task at greater than 80% success rate to in the lesion-affected visual field with a maximum luminance target (Michelson contrast 0.95).

40. Collecting the data to assess blindsight.
  a. Select a luminance contrast of the target (0.29° diameter) against the background luminance (1 cd/cm^2^) between 0 and 1.
    ***Note:*** The purpose of this step is to find the threshold of the target’s contrast at which the monkey can perform visually guided saccadic eye movements. At first, the contrast of the target should cover a wide range between 0 and 1.
    ***Note:*** The contrast should be chosen randomly in every trial so that the monkey cannot predict the target’s contrast in each trial.
  b. Reduce the range of the target’s contrast. An important point in this step is to estimate the threshold precisely. If you find a point at which the correct rate decreases (Figure 7A), reduce the range of the target’s contrast and focus on the point around which the correct rate suddenly drops to reveal the threshold.
    ***Note:*** If the monkey does not receive the reward frequently, it will stop performing the task. To maintain its motivation, a maximum contrast around 1 should always be included in the possible contrasts, and the probability that the chosen maximum contrast should be higher than that of the other low-contrast targets.

41. Constructing the deficit map.
  a. The sensitivity to luminance contrast is defined as the level of luminance contrast for the success ratio to be 0.79, which corresponds to d’ = 2 in the five-alternative forced choice method in signal detection theory (dotted line in Figure 7). The success ratio is plotted against luminance contrast.
  b. The threshold for luminance contrast is displayed at each target position (Figure 7B). This figure is constructed by plotting the detection threshold at each target location to show the extent of the visual field in which target detection is impaired.

**CRITICAL:** It is possible that you find the monkey can perform saccadic eye movement very correctly to a certain direction after the V1 lesion. There are two possible reasons. One is that the monkey performs a saccade to the direction when it cannot recognize direction of a visual target. Second is that the lesion size was small and intact visual field was left in the contra-lesional side (see problem 4).

**CRITICAL:** If you use the blindsight monkey for recording or pharmacological inactivation of the neural activity, it is strongly recommended to take MR images before starting your experiments (see problem 5).

### Post-mortem identification

**Timing: several weeks**

After all of the experiments are conducted, it is necessary to assess the lesioned brain area. For this assessment, *ex vivo* MRI and histological processing after perfusion are used. MR images allow us to reconstruct brain structure after lesioning, and histological processing provides us with more precise information about the lesioned area based on its cytoarchitecture. Furthermore, in general, the lateral geniculate nucleus (LGN) is significantly retrogradely degenerated after V1 lesioning. Furthermore, because a considerable number of neurons in the Konio-cellular layer (K-cells) project to the visual area MT,^8^ it has been proposed that K-cells survive V1 lesioning and mediate blindsight.^9^ Therefore, it is necessary to investigate how many K-cells survive at the end of the experiments. For this purpose, we routinely conduct immunohistochemistry against NeuN and CaMKII, which stain K-cells.

**Figure 8:**
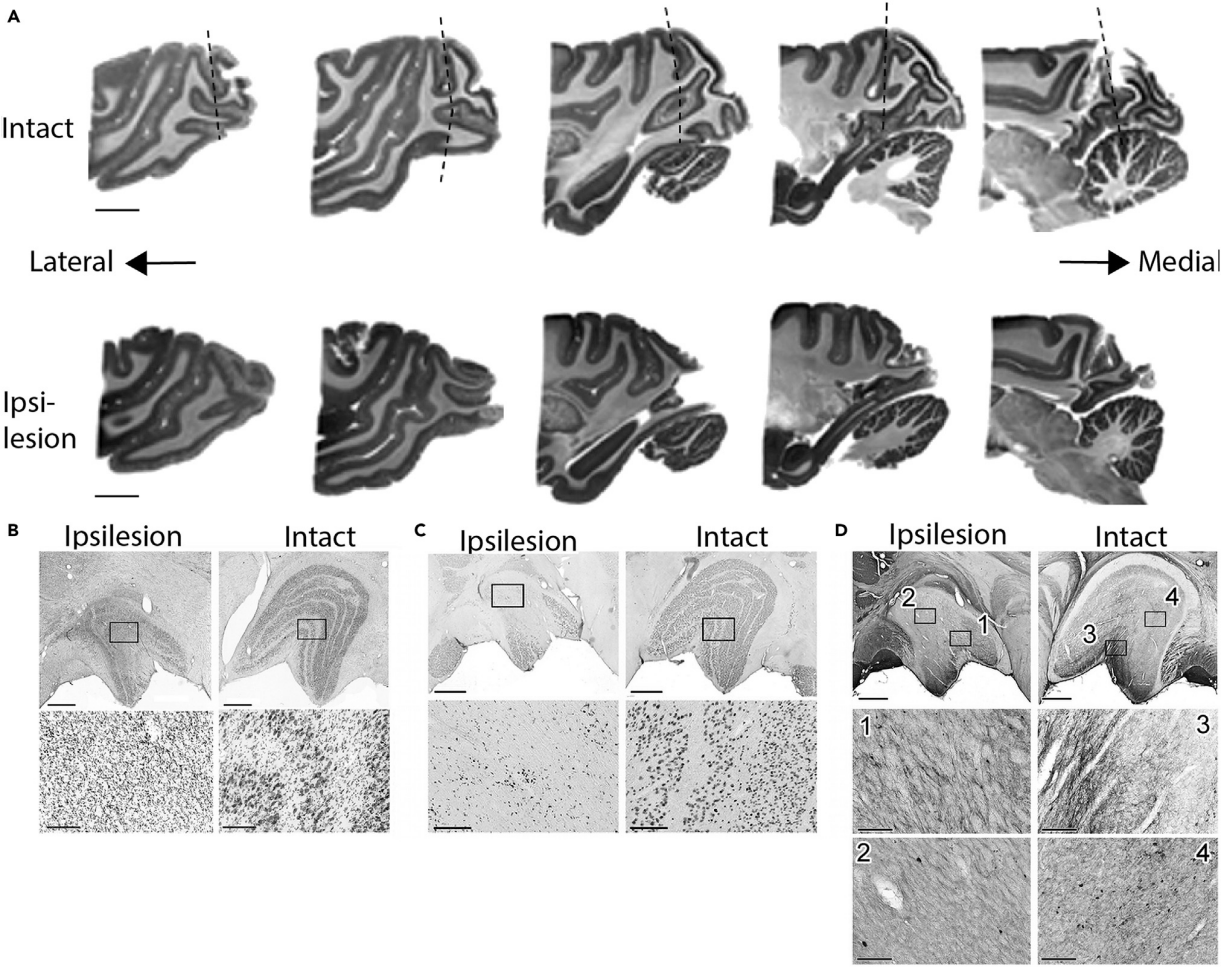
Post-mortem identification of the V1 lesion (A) Sagittal planes reconstructed from MR images. Upper panels show the intact side and lower panels show the ipsilesional side. The images are arranged from left to right in order from lateral to medial planes. Dashed lines in the upper panels indicate the border of V1. (B) Nissl-stained samples of the LGN in the ipsilesional side (left) and intact side. The area surrounded by the square is enlarged in the panel below. (C) LGN samples with anti-NeuN immunostaining. (D) LGN samples with anti-CaMKII immunohistochemistry. Scale bars; (A) 10 mm, (B–D) the first row, 1 mm, the second and third row, 200 μm, respectively. (B and C) Modified from Takakuwa et al.^2^
44. Histology 1: Confirmation of V1 lesioning by Nissl staining (Figure 8B).
  a. Replacement by sucrose. Put the brain blocks into 10% sucrose in 0.1 M PB, and wait until the brain sinks. Continue in 20% and 30% sucrose solution with the same protocol.
  b. Make 40-μm-thick frozen sections with a sliding microtome.
  c. Mount the sections on gelatin-coated glass slides.
  d. Cresyl violet staining.
    i. Incubate the slides in 70%, 80%, 90%, and 100% ethanol for 10 min each.
    ii. Incubate the slides in xylene for 2 h.
    iii. Remove the xylene by incubating the slides in 100%, 90%, 80%, 70%, and 50% ethanol and in tap water for 5 min each.
    iv. Stain in cresyl violet solution for 10 min.
    v. Differentiate the staining of Nissl bodies carefully in 30%, 50%, 70%, 90%, and 100% ethanol for 5 min each.
    vi. Incubate the slides in xylene for 2 h.
  e. Cover the sections with mounting medium and coverslips.
  f. Use a light microscope (BZ-X710 and BZ-X810; Keyence, Osaka, Japan) to observe and take photographs of the visualized neurons.
45. Histology 2: Confirmation of the degeneration of the LGN with anti-NeuN immunostaining (Figure 8C).
  Perform free-floating immunostaining as follows:
  a. Wash the tissue sections twice for 10 min in 0.3% (v/v) Triton X-100 in PBS (PBS-T) and incubate them in a solution of 0.6% H_2_O_2_ in Dent’s fixative followed by incubation in a blocking solution of 10% normal goat serum (NGS) in PBS-T for 1 h at 20°C–25°C.
  b. Incubate the sections with a mouse monoclonal anti-NeuN antibody (clone A60, 1:400, #MAB377; Millipore) in PBS-T/NGS at 4°C overnight (8–24 h).
  c. On the second day, rinse the sections four times in PBS-T.
  d. Incubate the sections for 2 h at 20°C–25°C with biotin goat anti-mouse IgG (1:200, #BA9200; Vector Laboratories) in PBS-T/NGS.
  e. After washing four times in PBS-T, incubate the sections in ABC-Elite solution (1:200; Vector Laboratories, CA, USA) in PBS-T for 1 h.
  f. After washing the sections with PBS, perform DAB staining with intensification using ammonium nickel (II) sulfate hexahydrate.
  g. Mount the sections on gelatin-coated glass slides and dehydrate them with the ethanol-xylene dehydration series mentioned above and coverslip the sections with mounting medium. Use a light microscope (BZ-X710 and BZ-X810; Keyence, Osaka, Japan) to observe and photograph the visualized neurons.
46. Histology 3: Confirmation of K-cells in the LGN with anti-CaMKII immunohistochemistry (Figure 8D).
  a. Wash the tissue sections for 10 min in 0.3% (v/v) Triton X-100 in PBS-T and incubate them in 50% (v/v) ethanol followed by incubation in a blocking solution of 0.05 M PBS with 2% NGS, 0.5% fish gelatin, 0.5% carrageenan, 0.02% NaN_3_, and 0.1% Triton X (PBS-T-2% NGS-fish gelatin/carrageenan) for 3 h at room temperature (15°C–25°C).
  b. Incubate the sections with 11.7 μg/mL mouse monoclonal anti-calmodulin-dependent protein kinase II alpha antibody (Thermo Fisher; Invitrogen CaMKII alpha monoclonal antibody [6G9]) in PBS-T-2% NGS-fish gelatin/carrageenan at 4°C overnight (8–24 h).
  c. On the second day, rinse the sections four times in PBS.
  d. Incubate the sections for 2 h with biotin goat anti-mouse IgG (1:100 [v/v], #BA9200; Vector Laboratories, CA, USA) in 0.05 M PBS with 2% NGS, 0.5% fish gelatin, 0.5% carrageenan, and 0.02% NaN_3_ at room temperature (15°C–25°C).
  e. After washing four times in PBS, incubate the sections in ABC-Elite solution (1:200; Vector Laboratories, CA, USA) in PBS-T for 1 h.
  f. After washing the sections with PBS, perform DAB staining (0.04% DAB with 0.04% NiCl_2_ and less than 0.003% H_2_O_2_ in Tris-buffered saline).
  g. The process after mounting with coverslips is the same as in step “Histology 2.”
  h. Count the number of K-cells in LGN sections using Fiji, which was included in ImageJ 28 (https://imagej.net/software/fiji).

***Note:*** Anti-CaMKII immunohistochemistry needs caution. One has to be careful about the perfusion of the animals, fixation of the brain tissue, thickness of the sections and preservation of the sections. Try to find the best condition for the reactions. Positive control is necessary. Carefully watch the reaction process for visualization with DAB under the microscope and try to set the reaction protocols to obtain reasonable contrast of the reaction products within 30 min. If the fixation of the brain tissue is not enough, it is recommended to put the sections in the 4% PFA solution for 30 min in the room temperature (15°C–25°C) and to wash carefully before activation with 50% ethanol, which sometimes can improve the quality of visualization.

42. Sacrifice the animal.
  a. In advance of the day of sacrifice, prepare the solutions (3–6 L of 0.05 M phosphate-buffered saline [PBS] and 5–7 L of 4% paraformaldehyde in 0.1 M PB).
  b. Introduce anesthesia. This step is the same as step 1 in the section “baseline magnetic resonance imaging (MRI).”
  c. Preparation in the dissection room.
    i. Insert an indwelling needle into the vein in the lateral and connect the indwelling needle to a tube that is connected to a syringe filled with extracellular fluid replacement solution.
    ii. Dissolve heparin (1 U/mL) in PBS.
    iii. Set a push-pull type ventilation system.
  d. Inject sodium thiopental (80 mg/kg) through the indwelling needle (i.v.). Ensure there are no reflexes (e.g., flexion reflex and/or pupillary light reflex).
  e. Cut the skin and muscles below the chest bone.
  f. Cut the chest bone with poultry scissors until the heart can be observed. Be careful not to cut the diaphragm.
  g. Peel off the pericardium to expose the heart.
  h. Make a small cut on the left ventricle of the heart with scissors.
  i. Insert a sonde through the small hole until it reaches the aorta and fix it with forceps.
  j. Turn on the peristaltic pump (100 mL/min) and flow the PBS. Start the push-pull type ventilation system.
  k. Cut the right heart ear with scissors.
  l. Flow the PBS until the blood becomes transparent (4–5 L).
  m. Switch to 4% paraformaldehyde and leave it until the animal stops trembling (4–5 L, 0.5–0.8 L/kg).
  n. Remove the brain from the skull.
43. MRI of *ex vivo* specimen (Figure 8A).
  a. Immerse the brain in a solution containing a gadolinium-based MR contrast agent (2 mM), and perform imaging after replacing the fluid with a proton-free magnetic susceptibility matching fluid (Fluorinert FC-43; 3M Company, MN, USA) and vacuuming the air inside the brain.
  b. Perform *ex vivo* imaging using an investigational whole-body human 7T scanner (MAGNETOM 7T; Siemens Healthineers, Erlangen, Germany).^10^
  c. Obtain images using insertable inductively coupled volume coils with a diameter of 64 mm with a 3D gradient-echo sequence in isotropic 160 μm resolution (FOV = 72 mm, TR/TE = 200/20 ms, bandwidth = 30 Hz/pixel).
  d. Realign the brain to the stereotaxic position as in the *in vivo* condition and then re-slice it using imaging software (PMOD Technologies, Zürich, Switzerland) (Methods videos S1 and S2).

Methods video S1. 3D brain image in lesion side constructed by MRI of *ex vivo* specimen, related to step 43

Methods video S2. 3D brain image in intact side constructed by MRI of *ex vivo* specimen, related to step 43

## Expected outcomes

### Assessment of visuomotor and cognitive functions

In the above, the process to establish a monkey with blindsight was described. Then, we move on to the main experiments to test the visuomotor and cognitive functions and the underlying neural circuit mechanisms by combining a variety of techniques using neuroimaging, electrophysiology, reversible inactivation, pathway-selective manipulation by viral vectors, psychophysics, immunohistochemistry, and etc. in a variety of visuomotor and cognitive task settings. Please refer to our recent review article^7^ for details.

## Limitations

How well do our blindsight model monkeys mimic human blindsight patients? The visual responses of the blindsight model look very similar to those of human patients. For example, they can respond to visual targets after lesioning, and the sensitivity to luminance contrast is clearly lower than in the intact visual field. A major concern is whether the visual awareness of V1-lesioned monkeys is as impaired as that of human blindsight patients. As it is not possible to obtain verbal reports about the subject’s cognition in nonhuman animals, we have to assess their cognitive status indirectly. Regarding the visual awareness of V1-lesioned monkeys, Cowey and Steorig^11^ showed that they have difficulty in reporting their “seeing” experience in the Yes-No task setting; however, there were criticisms about their task design.^12^^,^^13^ For example, different stimulus conditions were used between the forced-choice and Yes-No choice tasks and decision bias based on signal detection theory was ignored in the study of Cowey and Steorig.

Considering such criticisms, our group designed another Yes-No choice task experiment using the same stimulus settings in both the forced-choice and Yes-No choice studies and applied signal detection theory to remove the factors influenced by the subject’s bias.^12^ We found that sensitivity was reduced in the blindsight condition, which was similar to the blindsight patient GY. Therefore, we concluded that visual awareness was impaired, but not perfectly, in our blindsight monkeys, which mimic “type II blindsight” patients like GY, who mentions “feeling something (but not seeing)” for visually captured objects.

Conversely, the performance level of the blindsight model is higher than that of human patients, which might be because of our training regimen. Our blindsight model monkeys start their training immediately after the recovery period from lesion surgery. Additionally, we tend to use young monkeys. It is possible that these components are critical for the higher performance level of our blindsight monkeys.

## Troubleshooting

### Problem 1

The monkey stops its training or forgets what it learned (step 18).

### Potential solution

Go back to the previous step of training. If a monkey cannot understand what is required, it easily loses its motivation and stops its behavior. In this case, use the previous training step that the monkey knows well and increase its motivation. After that, start the next training step.

### Problem 2

Infection occurred after V1 lesioning (step 37).

### Potential solution

It is difficult to eliminate the risk of infection completely because the skin is not closed. Cleaning with Ringer’s solution should be performed frequently (e.g., once a day).

### Problem 3

The monkey can perform saccadic eye movements to the large portion of the lesion-affected visual field immediately after V1 lesioning before starting post-lesion training (step 38).

### Potential solution

Additional surgery is necessary. In this case, it is possible that V1 is not lesioned sufficiently. Especially, the lateral part of V1 might be intact if the monkey can make saccades toward visual targets at 5°–15° eccentricity.

### Problem 4

The monkey can perform saccadic eye movements to a particular location in the lesion-affected visual field (step 41).

### Potential solution

Presenting the visual stimulus in the visual field where luminance sensitivity is high in future tests should be avoided.

### Problem 5

Neuronal recordings or microinjection of agents does not work as expected (step 41).

### Potential solution

In many cases, the brain tissue gradually becomes dislocated on the ipsilesional side due to emergence of the empty intracranial space after removing the V1. Therefore, it is recommended to take the structural MRI images again before these experiments.

## Resource availability

### Lead contact

Further information and requests for resources and reagents should be directed to and will be fulfilled by the technical contact, Norihiro Takakuwa (norihiro.takakuwa@brain.mpg.de), by the lead contact, Tadashi Isa (isa.tadashi.7u@kyoto-u.ac.jp).

### Materials availability

Most of the materials used in this protocol are available commercially. Our chamber is designed so a Crist grid (6-YGD-D1; Crist Instrument) can be inserted. If you have a plan to use such an extra grid in your future experiments, change the design.

## Data Availability

This study did not generate any unique data sets or code.
